# Supplementary material for: Afadin sorts different retinal neuron types into accurate cellular layers
Source: eLife. 2026 Jan 14;14:RP105575. doi: 10.7554/eLife.105575 (PMC12803510; doi:10.7554/eLife.105575)
Supplement: Figure 4—source data 2. [file elife-105575-fig4-data2.pdf]

AfadincKO-Retina ERG Datasets

diagnosys
leading the wave

Patient: AFACKO-7 DOB:1/26/2019 Female Investigator :LDS+HG
Examiner: SYSDBA **Date Tested:** 2/27/2019 (Updated: 3/1/2019) **Time Tested:** 1:39:45 PM
Unique ID: 11B021A1-8F86-48BF-ABE6-A350-A212-6D6D **Dark Adapted Intensity Series LDS - Afadin [14803-C || ECN 1011 || 29 September 2017]**

1 - 3 cd.s/m² White

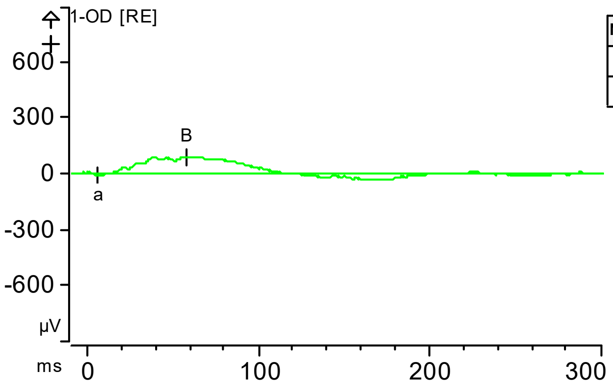

Name	uV	ms
a	-11.7	7
B	102.8*	58

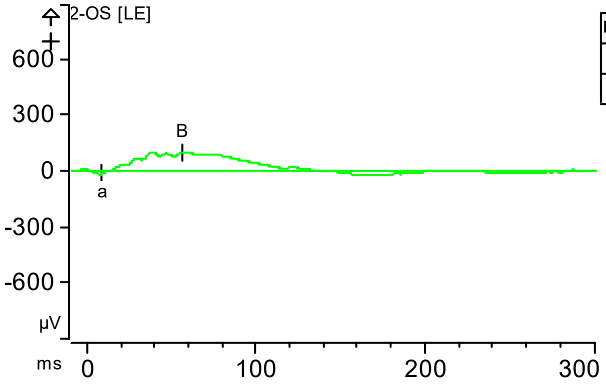

Name	uV	ms
a	-16.96	10
B	117.8*	58.5

2 - 10 cd.s/m² White

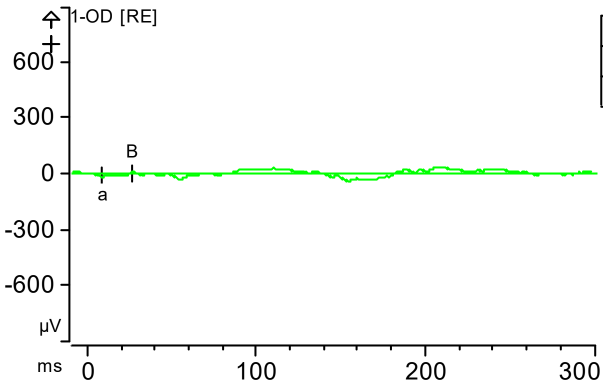

Name	uV	ms
a	-18.43	10.5
B	26.29*	28.5

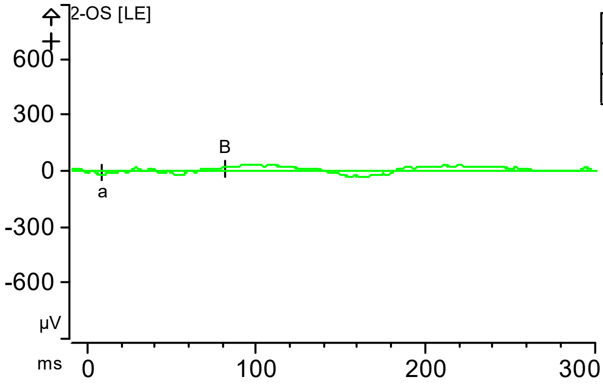

Name	uV	ms
a	-19.7	10.5
B	41.42*	83

3 - 30 cd.s/m² White

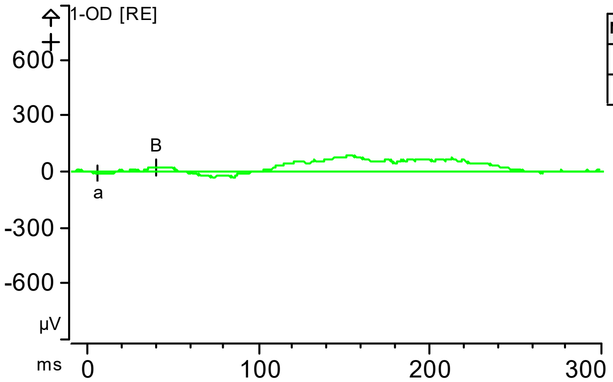

Name	uV	ms
a	-14.33	6
B	40.95*	41

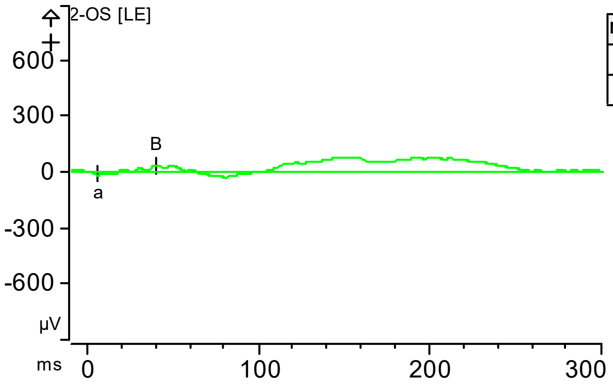

Name	uV	ms
a	-18.92	7
B	56.8*	41

4 - 100 cd.s/m² White

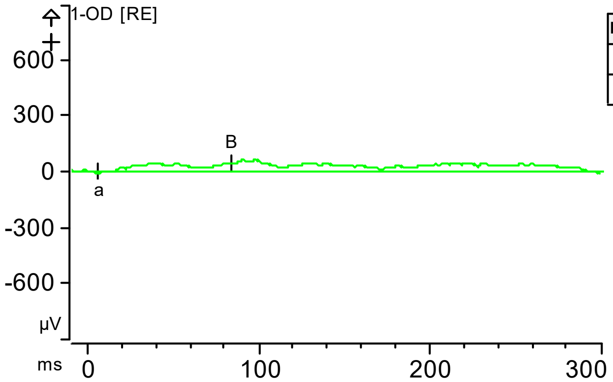

Name	uV	ms
a	-6.359	7
B	54.97*	85

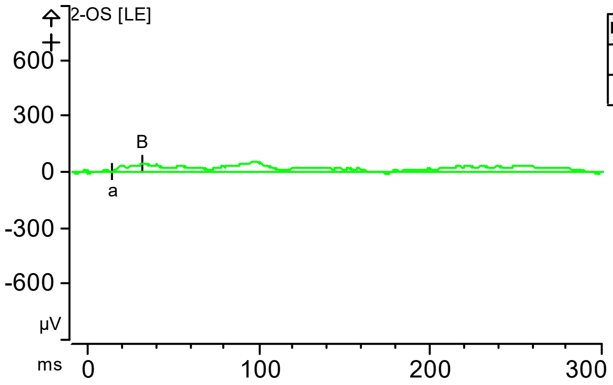

Name	uV	ms
a	-0.28	15
B	47.19*	33

Patient: AFACKO-2 DOB:1/26/2019 Male Investigator :LDS+HG

Examiner: SYSDBA **Date Tested:** 2/27/2019 (Updated: 3/1/2019) **Time Tested:** 11:22:12 AM

Unique ID: 8F311B0D-0D13-4FF1-99F7-5DAE-A2B3-0E49 **Photopic Intensity Ramp LDS - Afadin [14803-C || ECN 1011 || 29 September 2017]**

1 - 1 cd.s/m² White

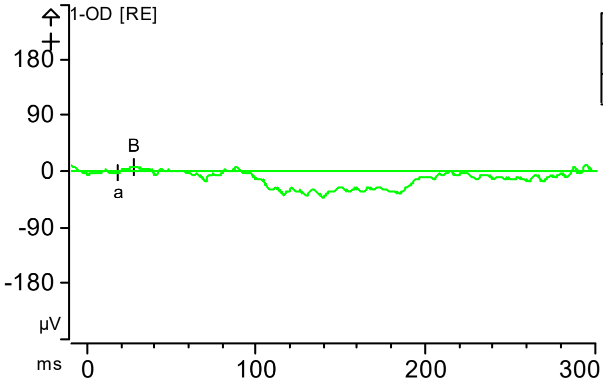

Name	uV	ms
a	-4.08	19.5
B	11.69*	29.5

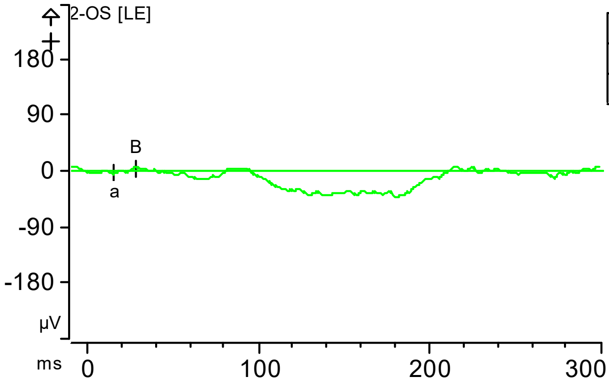

Name	uV	ms
a	-4.942	16
B	10.52*	29

2 - 3 cd.s/m² White

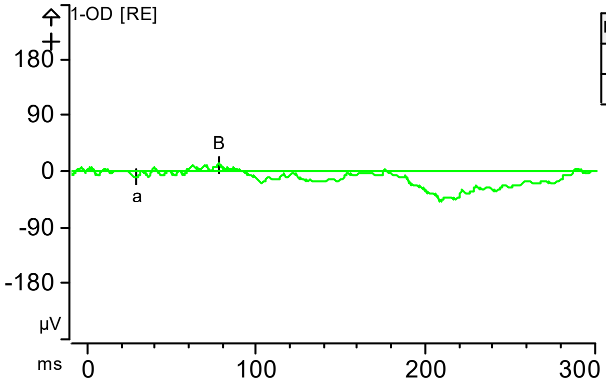

Name	uV	ms
a	-10.64	30.5
B	22.52*	79.5

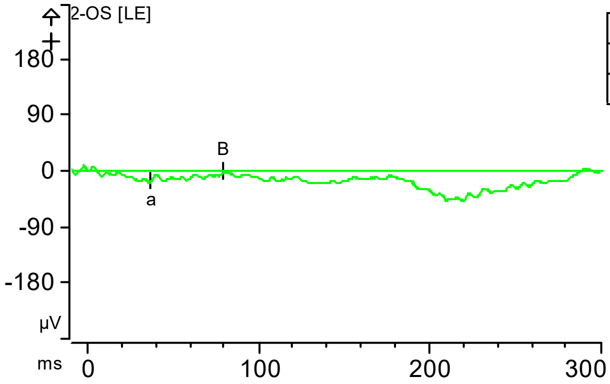

Name	uV	ms
a	-18.87	37
B	16.17*	80

3 - 10 cd.s/m² White

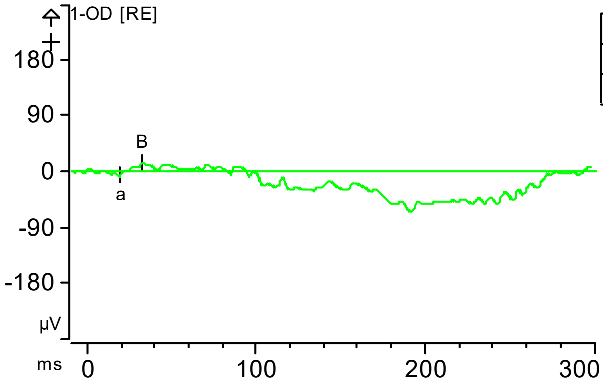

Name	uV	ms
a	-9.377	21
B	22.51*	34.5

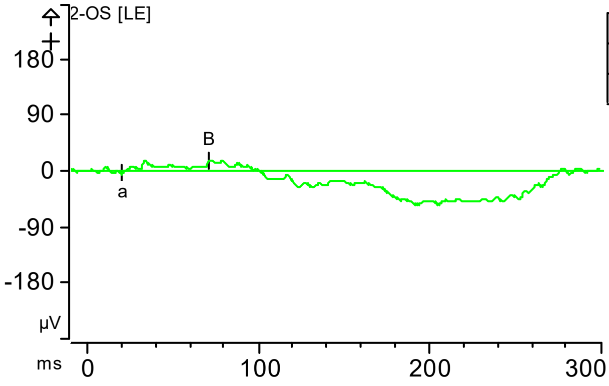

Name	uV	ms
a	-5.381	21
B	22.59*	72

4 - 30 cd.s/m² White

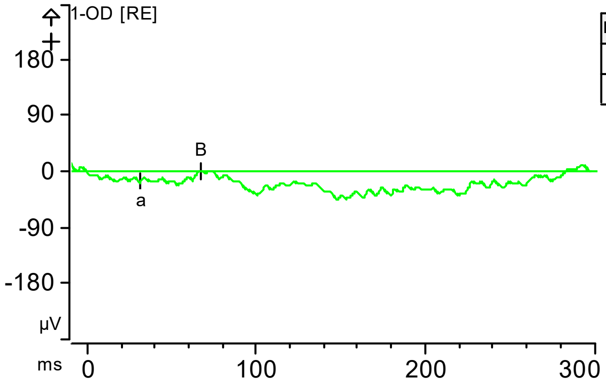

Name	uV	ms
a	-19.51	32.5
B	20.3*	68.5

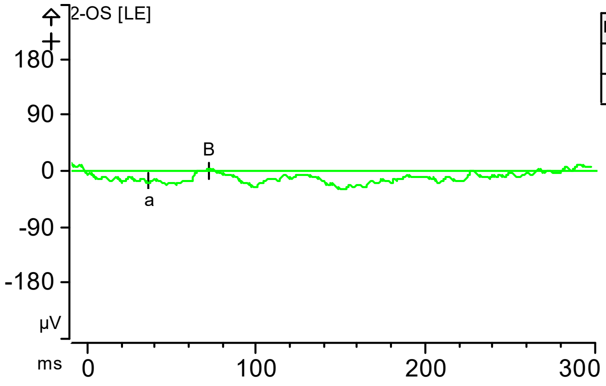

Name	uV	ms
a	-19.2	38
B	21.89*	73.5

Patient: AFACKO-2 DOB:1/26/2019 Male Investigator :LDS+HG

Examiner: SYSDBA **Date Tested:** 2/27/2019 (Updated: 3/1/2019) **Time Tested:** 11:22:12 AM

Unique ID: 8F311B0D-0D13-4FF1-99F7-5DAE-A2B3-0E49 **Photopic Intensity Ramp LDS - Afadin [14803-C || ECN 1011 || 29 September 2017]**

5 - 100 cd.s/m² White

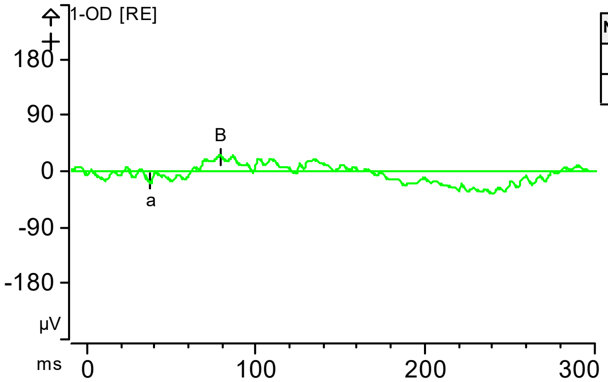

Name	uV	ms
a	-18.18	39.5
B	44.17*	80.5

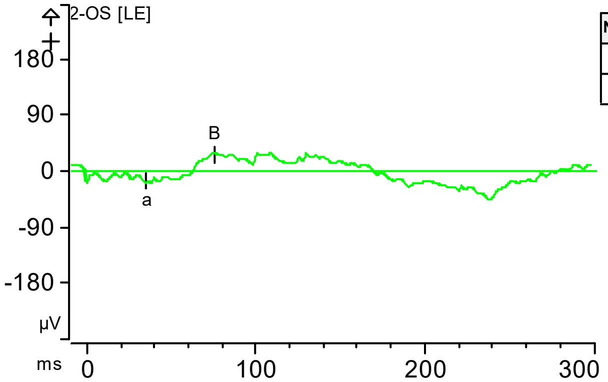

Name	uV	ms
a	-18.91	36.5
B	47.57*	77.5

AfadinWT-Retina ERG Datasets

diagnosys
leading the wave

Patient: AFAWT-9 DOB:1/26/2019 Female Investigator :LDS+HG
Examiner: SYSDBA **Date Tested:** 2/27/2019 (Updated: 3/1/2019) **Time Tested:** 1:55:19 PM
Unique ID: 6D25E9C3-33D7-4E6A-BEC4-D0D5-EC35-EE47 **Dark Adapted Intensity Series LDS - Afadin [14803-C || ECN 1011 || 29 September 2017]**

1 - 3 cd.s/m² White

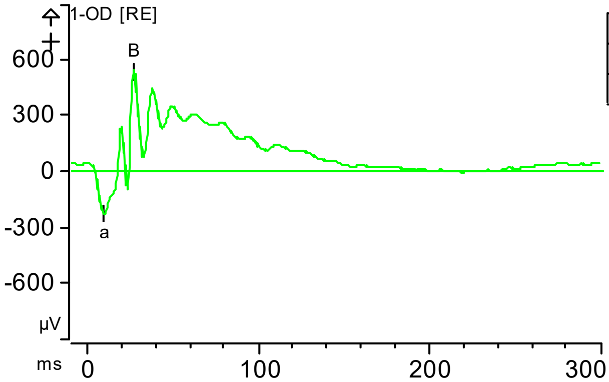

Name	uV	ms
a	-232.2	10
B	774.5*	28

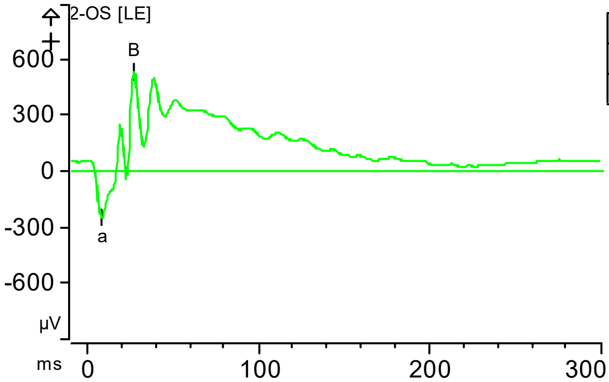

Name	uV	ms
a	-253.7	9
B	790.5*	28

2 - 10 cd.s/m² White

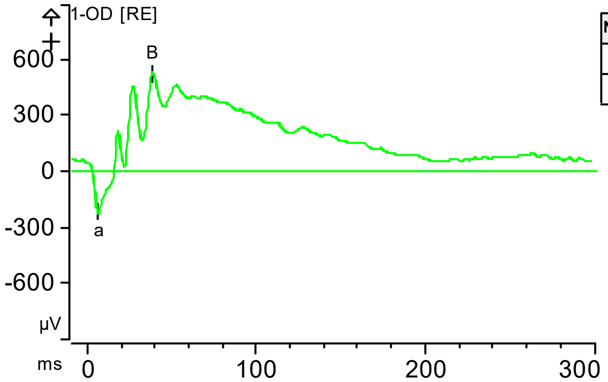

Name	uV	ms
a	-227.8	8
B	756.9*	40.5

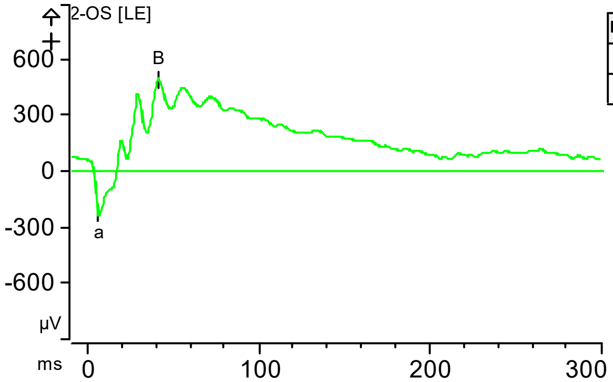

Name	uV	ms
a	-235.5	7
B	732.8*	42

3 - 30 cd.s/m² White

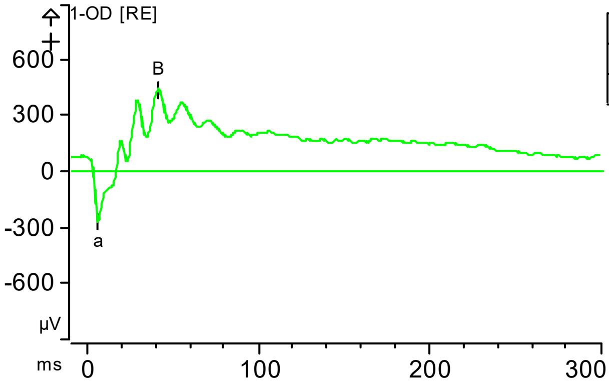

Name	uV	ms
a	-273.3	6.5
B	718.6*	42

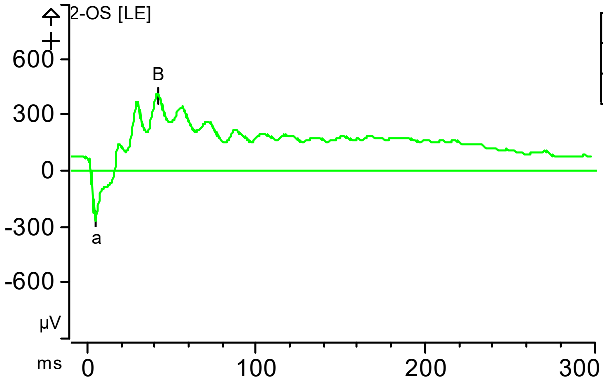

Name	uV	ms
a	-268.7	6.5
B	681.4*	43.5

4 - 100 cd.s/m² White

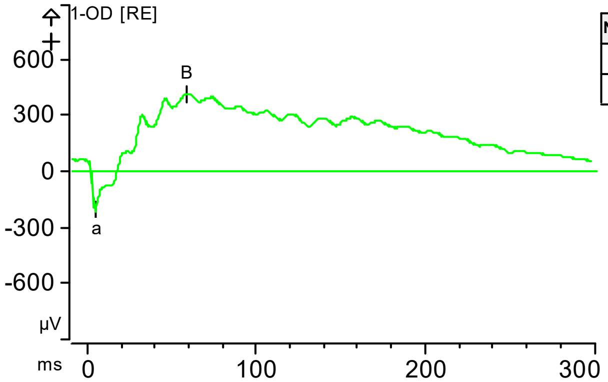

Name	uV	ms
a	-214.8	6.5
B	633.2*	60.5

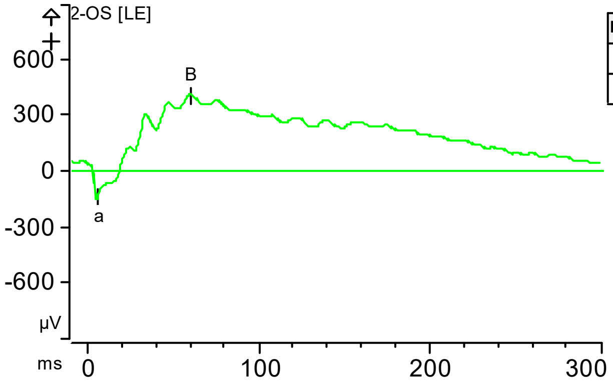

Name	uV	ms
a	-145.9	6.5
B	556.7*	61

diagnosys
leading the wave

Patient: AFAWT-9 DOB:1/26/2019 Female Investigator :LDS+HG
Examiner: SYSDBA **Date Tested:** 2/27/2019 (Updated: 3/1/2019) **Time Tested:** 2:00:44 PM
Unique ID: 7FD12961-901F-460F-86C5-41C1-2384-B9C5 **Photopic Intensity Ramp LDS - Afadin [14803-C || ECN 1011 || 29 September 2017]**

1 - 1 cd.s/m² White

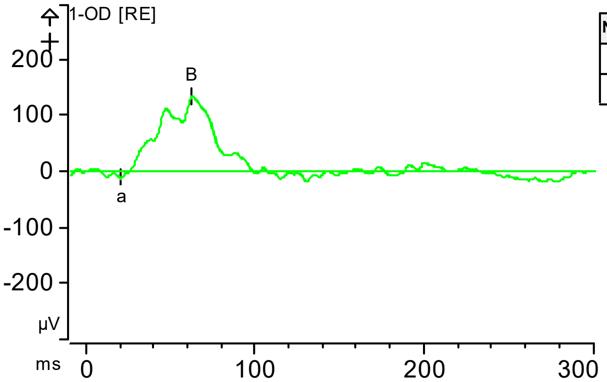

Name	uV	ms
a	-12.83	22
B	147.8*	64.5

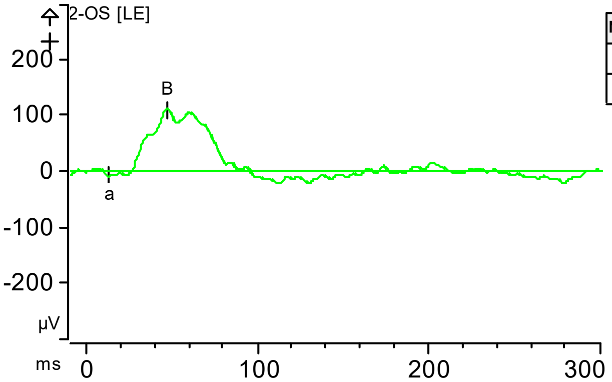

Name	uV	ms
a	-9.365	14
B	121.5*	48

2 - 3 cd.s/m² White

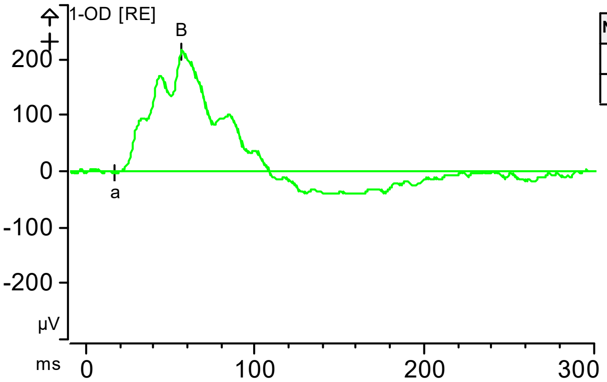

Name	uV	ms
a	-4.152	18.5
B	220.8*	58.5

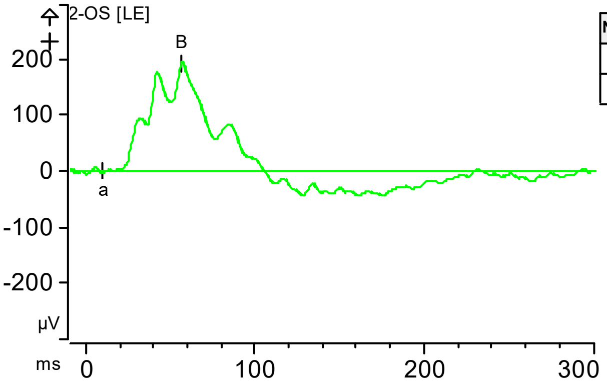

Name	uV	ms
a	-2.785	11
B	198.2*	58.5

3 - 10 cd.s/m² White

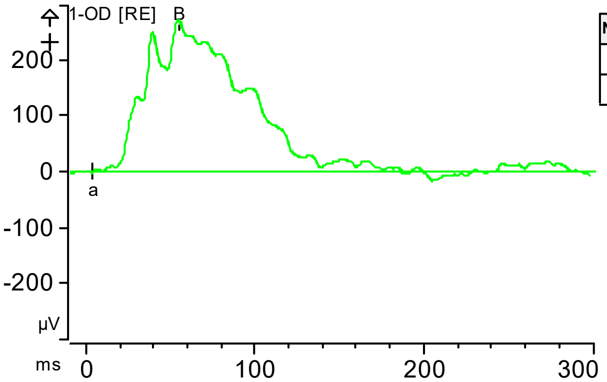

Name	uV	ms
a	-1.938	5
B	273.5*	56.5

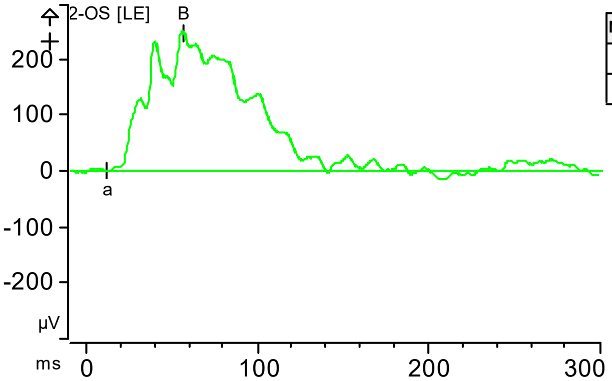

Name	uV	ms
a	-0.494	13
B	250.9*	57

4 - 30 cd.s/m² White

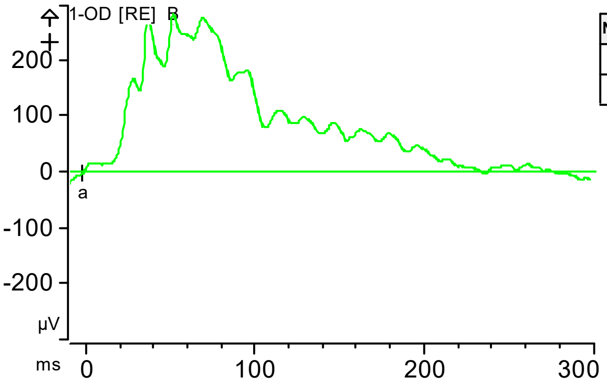

Name	uV	ms
a	-4.56	-1.5
B	286.5*	53.5

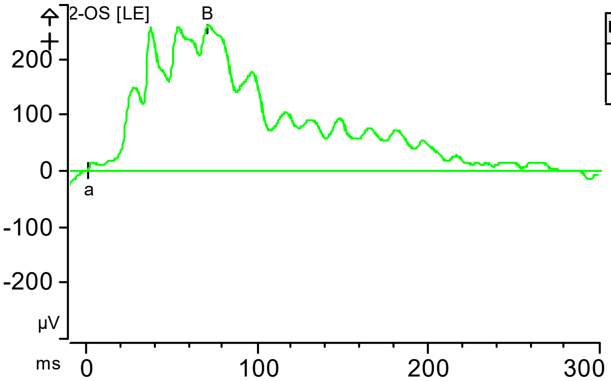

Name	uV	ms
a	-0.38	1.5
B	262.2*	72

Patient: AFAWT-9 DOB:1/26/2019 Female Investigator :LDS+HG
Examiner: SYSDBA **Date Tested:** 2/27/2019 (Updated: 3/1/2019) **Time Tested:** 2:00:44 PM
Unique ID: 7FD12961-901F-460F-86C5-41C1-2384-B9C5 **Photopic Intensity Ramp LDS - Afadin** **[14803-C || ECN 1011 || 29 September 2017]**

5 - 100 cd.s/m² White

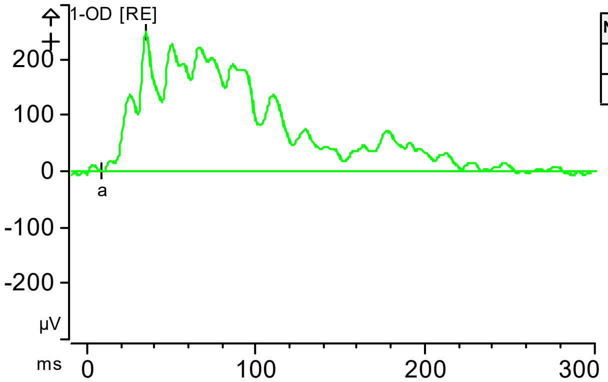

Name	uV	ms
a	-0.876	10.5
B	253.2*	36.5

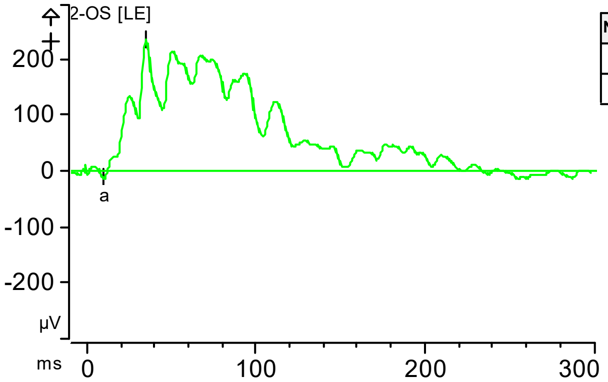

Name	uV	ms
a	-12.95	11.5
B	251.1*	36.5
